# Integrative machine learning and network toxicology framework for assessing environmental pollutant TCDD-induced osteoarthritis risk: a computational cheminformatics approach

**DOI:** 10.3389/fphar.2026.1811221

**Published:** 2026-05-28

**Authors:** Lei Li, Yihong Zhang, Wenkui Qiu, Xiaolong Li, Jianjun Ji, Lichao Cao, Jiaxing Lü, Xuejin Zhu, Zhenyan Su

**Affiliations:** Department of Orthopedics, Kaifeng Central Hospital Affiliated to Xinxiang Medical University, Kaifeng, Henan, China

**Keywords:** environmental pollutants, machine learning, molecular docking, network toxicology, osteoarthritis, TCDD

## Abstract

**Objective:**

Osteoarthritis (OA) is a prevalent degenerative joint disease influenced by both genetic susceptibility and environmental factors. Increasing evidence suggests that exposure to persistent environmental pollutants, such as 2,3,7,8-tetrachlorodibenzo-p-dioxin (TCDD), may contribute to OA progression; however, the molecular mechanisms underlying these effects remain unclear. Therefore, this study aimed to elucidate the potential molecular interactions and mechanisms by which TCDD may influence OA development using an integrated systems-level computational strategy.

**Methods:**

OA-related genes were identified by integrating multiple transcriptomic datasets from the Gene Expression Omnibus (GEO) database through differential expression analysis and weighted gene co-expression network analysis (WGCNA). Putative TCDD targets were collected from public chemical–protein interaction databases, and overlapping targets were identified to construct a disease–toxicant interaction network. Gene Ontology (GO) and Kyoto Encyclopedia of Genes and Genomes (KEGG) enrichment analyses were performed to explore the biological functions and pathways involved. Key OA-related proteins associated with TCDD exposure were further prioritized using an integrative machine learning framework. Immune cell infiltration analysis was conducted to investigate associations between hub proteins and the OA immune microenvironment. Finally, molecular docking and molecular dynamics simulations were employed to evaluate the stability and binding behavior of TCDD with the identified key proteins.

**Results:**

A total of 471 OA-related genes were identified, among which 43 overlapped with predicted TCDD targets. Functional enrichment analysis indicated that these targets were primarily involved in inflammatory signaling, arachidonic acid metabolism, calcium signaling, and G protein-coupled receptor–related pathways. Machine learning analysis identified four hub proteins—PTGS1, CBR1, HTR2B, and PTGS2—with robust diagnostic relevance across independent cohorts. Immune infiltration analysis revealed that these hub proteins were significantly associated with macrophage polarization, mast cell activation, and T-cell dysregulation in OA. Molecular docking and molecular dynamics simulations demonstrated stable binding conformations between TCDD and all four hub proteins, with trajectory analyses confirming persistent ligand binding and structural stability throughout the simulations.

**Conclusion:**

PTGS1, CBR1, HTR2B, and PTGS2 were identified as key hub proteins potentially mediating TCDD-associated osteoarthritis. Our integrated computational framework highlights specific inflammatory, metabolic, and neurotransmitter pathways linking environmental pollutant exposure to OA pathogenesis, providing actionable targets for future experimental validation.

## 1 Introduction

Osteoarthritis (OA) is the most prevalent degenerative joint disease worldwide, affecting approximately 595 million individuals globally (GBD 2021 Osteoarthritis Collaborators, 2023). The disease is characterized by cartilage degradation, synovial inflammation, and subchondral bone remodeling, resulting in chronic pain and functional disability (Martel-Pelletier et al., 2016). Beyond traditional risk factors such as aging and mechanical stress, environmental pollutant exposure has emerged as a potential contributor to OA pathogenesis (Meng et al., 2024).

2,3,7,8-Tetrachlorodibenzo-p-dioxin (TCDD) is among the most toxic environmental pollutants, primarily generated through industrial processes and cigarette smoking (Van den Berg et al., 2006). TCDD exerts its toxic effects through binding to the aryl hydrocarbon receptor (AhR), subsequently activating downstream signaling pathways involved in inflammation and cellular stress responses (Mimura and Fujii-Kuriyama, 2003). Studies have demonstrated that TCDD impairs chondrocyte proliferation and differentiation, induces cartilage matrix degradation, and promotes inflammatory cytokine expression (Burns et al., 2015; Kobayashi et al., 2008). Epidemiological evidence has also revealed positive associations between dioxin exposure and arthritis prevalence (Guo et al., 1999). However, the systematic molecular mechanisms underlying TCDD-induced effects on OA remain unclear.

Network toxicology integrates systems biology and bioinformatics to systematically elucidate multi-target toxicity mechanisms (Valls-Margarit et al., 2023). In this study, we employed network toxicology combined with machine learning algorithms to investigate the potential toxic mechanisms of TCDD on OA. By integrating multiple GEO datasets, we identified OA-related genes through differential expression analysis and weighted gene co-expression network analysis (WGCNA). Hub genes were screened using machine learning approaches and validated through molecular docking simulations. Our findings provide novel insights into the molecular mechanisms by which TCDD may contribute to OA pathogenesis.

## 2 Methods

### 2.1 Data acquisition and preprocessing

Gene expression datasets related to osteoarthritis were retrieved from the Gene Expression Omnibus (GEO) database (https://www.ncbi.nlm.nih.gov/geo/). For the training cohort, four microarray datasets were included: GSE55457 (GPL96 platform, n = 46), GSE82107 (GPL570 platform, n = 17), GSE206848 (GPL570 platform, n = 14), and GSE55235 (GPL96 platform, n = 46), comprising a total of 123 samples (54 controls and 69 OA samples).

To eliminate batch effects among the training datasets from different platforms, the ComBat algorithm from the sva R package was applied (Leek et al., 2012). The effectiveness of batch correction was evaluated by principal component analysis (PCA) and visualization of expression distribution using box plots. After batch correction, samples from different datasets showed homogeneous expression distributions and were well-integrated in PCA plots, indicating successful removal of technical variations while preserving biological signals. Furthermore, to quantitatively assess the batch correction efficacy beyond visual inspection, Principal Variance Component Analysis (PVCA) was performed to calculate the variance proportions attributed to batch and biological effects before and after correction.

### 2.2 Identification of differentially expressed genes and WGCNA

Differential expression analysis was performed using the limma R package (Ritchie et al., 2015). A linear model was fitted to the batch-corrected expression data with a design matrix specifying the group factor (Control vs. OA). Empirical Bayes moderation was applied using the eBayes function to obtain moderated t-statistics and P-values. The P-values were adjusted for multiple testing using the Benjamini-Hochberg false discovery rate (FDR) method. Genes were considered significantly differentially expressed if they met the following criteria: |log_2_ fold change (log_2_FC)| > 0.585 (corresponding to a 1.5-fold change) and adjusted P-value <0.05. The results were visualized using volcano plots generated with the ggplot2 R package, and heatmaps of the top DEGs were generated using the pheatmap R package with row-wise z-score normalization.

### 2.3 Weighted gene co-expression network analysis

To identify gene modules associated with OA phenotype, WGCNA was performed using the WGCNA R package (Langfelder and Horvath, 2008). Genes with low expression variability were filtered out to reduce noise. Sample clustering was performed using average linkage hierarchical clustering based on Euclidean distance, and outlier samples were identified and removed. The soft-thresholding power (β) was determined using the pickSoftThreshold function by analyzing the scale-free topology fit index across a range of powers (1–20). The power at which the scale-free topology fit index (R^2^) first reached 0.8 was selected. Using the selected soft-thresholding power, a signed weighted correlation network was constructed, and gene modules were identified using the dynamic tree cut algorithm with a minimum module size of 60 genes. Modules with highly correlated eigengenes (correlation >0.75) were merged.

Module eigengenes (MEs) were calculated to represent the overall expression pattern of each module. The correlation between MEs and clinical traits (Control vs. OA) was assessed using Pearson correlation analysis. Modules with significant correlations (|r| > 0.3 and P < 0.05) with OA status were considered disease-associated modules. The union between DEGs and WGCNA module genes was determined to identify candidate genes for subsequent analysis.

### 2.4 Prediction of TCDD targets and identification of common targets

The molecular targets of TCDD were predicted using three complementary databases: ChEMBL (https://www.ebi.ac.uk/chembl/) (Mendez et al., 2019), SwissTargetPrediction (http://www.swisstargetprediction.ch/) (Daina et al., 2019) and SEA (https://sea.bkslab.org/) (Keiser et al., 2007). The SMILES structure of TCDD (ClC1 = CC2 = C(C=C1Cl)OC3 = CC(Cl) = C(Cl)C=C3O2) was used as input. Predicted targets from the three databases were merged and deduplicated to obtain a comprehensive list of TCDD-related targets. OA-related genes were defined as the intersection of DEGs and genes from disease-associated WGCNA modules. The common targets between TCDD and OA-related genes were identified using Venn diagram analysis.

### 2.5 Functional enrichment analysis

Gene Ontology (GO) and Kyoto Encyclopedia of Genes and Genomes (KEGG) pathway enrichment analyses were performed using the clusterProfiler R package (Wu et al., 2021). Gene symbols were converted to Entrez IDs using the org.Hs.eg.db annotation package. For GO analysis, all three categories (Biological Process, Cellular Component, and Molecular Function) were analyzed. Enriched terms were considered statistically significant with P-value <0.05 and adjusted P-value (Benjamini-Hochberg correction) < 0.05. The results were visualized using bar plots, bubble plots, and circos plots generated with the ggplot2, enrichplot, and circlize R packages.

### 2.6 Machine learning-based feature selection

To identify the most predictive biomarkers from the common targets, a comprehensive machine learning framework integrating 127 algorithmic combinations was employed (Liu Z. et al., 2022). The framework combined various feature selection methods with classification algorithms, including Elastic Net (Enet) with different alpha values (0.1–1.0), Least Absolute Shrinkage and Selection Operator (LASSO), Ridge regression, stepwise logistic regression (forward, backward, and both directions), Support Vector Machine (SVM), Linear Discriminant Analysis (LDA), gradient boosting machine (glmBoost), partial least squares regression for generalized linear models (plsRglm), Random Forest (RF), Gradient Boosting Machine (GBM), XGBoost, and Naive Bayes.

The merged training dataset was used for model construction, while GSE169077 and GSE178557 served as independent external validation sets. Expression data were centered and scaled before model training. For each algorithm, 10-fold cross-validation was performed to optimize hyperparameters. Model performance was evaluated using area under the receiver operating characteristic curve (AUC) across all datasets. The optimal model was selected based on the highest mean AUC. The selected features (genes) from the optimal model were extracted as hub genes for subsequent analysis.

To interpret the contribution of each feature to the model predictions, SHapley Additive exPlanations (SHAP) analysis was performed. SHAP values were calculated using the permutation-based method implemented in the kernelshap R package. The SHAP values were visualized using the shapviz R package, including bar plots for overall feature importance (mean absolute SHAP values), beeswarm plots for feature value distributions and their impact on predictions, dependence plots for individual feature effects, and waterfall plots for single prediction explanations. To strictly mitigate the risk of overfitting during the evaluation of the 127 algorithm combinations, 10-fold cross-validation was implemented within the training set, and model generalization was universally verified across two independent external cohorts. Furthermore, to quantitatively assess the stability of the machine learning framework, a feature selection frequency analysis was conducted by calculating the percentage of times each target gene was selected across all executed algorithmic combinations.

### 2.7 Immune infiltration analysis and hub gene–immune cell correlation

The relative proportions of 22 immune cell types in each sample were estimated using the CIBERSORT algorithm, a deconvolution method based on support vector regression (SVR). The LM22 signature matrix containing 547 marker genes distinguishing 22 human immune cell subtypes was used as reference. Input gene expression data were quantile-normalized (QN = TRUE), and 1,000 permutations were performed to calculate statistical significance. Samples with CIBERSORT P-value <0.05 were retained for subsequent analysis.

Spearman correlation coefficients were calculated between all pairs of the 22 immune cell types based on their estimated proportions in OA samples. Correlation coefficients and associated P-values were visualized in a heatmap using the corrplot R package. P-values were adjusted using the Benjamini–Hochberg false discovery rate (FDR) method. Spearman correlation analysis was performed between the expression levels of the four hub genes (PTGS1, CBR1, HTR2B, PTGS2) and the estimated proportions of 22 immune cell types. Correlation coefficients and FDR-adjusted P-values were calculated. Significant correlations were defined as |r| > 0.3 and FDR <0.05. Results were visualized in a heatmap and a lollipop plot using ggplot2.

Scatter plots were generated for representative significant gene–immune cell pairs to visually validate correlation trends. Regression lines, correlation coefficients (r), and FDR values were annotated on each plot using ggplot2. Differences in immune cell proportions between OA and control groups were assessed using the Wilcoxon rank-sum test. P-values <0.05 were considered statistically significant. Results were visualized using box plots, with asterisks indicating significance levels (*P < 0.05, **P < 0.01, ***P < 0.001).

### 2.8 Molecular docking

Molecular docking was performed to evaluate the binding potential between TCDD and the identified hub target proteins using CB-Dock2 (https://cadd.labshare.cn/cb-dock2/) (Liu Y. et al., 2022), a cavity-detection guided blind docking web server. The three-dimensional structure of TCDD was obtained from the PubChem database (CID: 15625). The crystal structures of the target proteins were retrieved from the RCSB Protein Data Bank (PDB).

CB-Dock2 automatically identifies binding sites using a curvature-based cavity detection approach and performs molecular docking using AutoDock Vina (Trott and Olson, 2010). The docking results were ranked by binding affinity (kcal/mol), with more negative values indicating stronger binding interactions. A binding affinity of less than −5.0 kcal/mol was generally considered indicative of good binding potential, while values below −7.0 kcal/mol suggested strong binding interactions. The key residues involved in protein-ligand interactions were identified and visualized using PyMOL software.

### 2.9 Molecular dynamics simulation and trajectory analysis

Molecular dynamics (MD) simulations were performed using GROMACS to evaluate the stability of the protein–TCDD complexes. The initial complex structures were obtained from molecular docking results. Each complex was placed in a cubic simulation box with a minimum distance of 1.0 nm between the solute and the box boundary using the editconf module. The systems were solvated with SPC water molecules, and counterions (Na^+^ and Cl^−^) were added to neutralize the total charge.

Energy minimization was carried out using the steepest descent algorithm to remove unfavorable contacts. Subsequently, the systems were equilibrated in two steps: NVT equilibration to stabilize the temperature, followed by NPT equilibration to stabilize the pressure and density. During the equilibration stages, position restraints were applied to the protein and ligand atoms. After equilibration, production MD simulations were performed under NPT conditions.

Trajectory processing was conducted to remove periodic boundary condition (PBC) artifacts by reconstructing whole molecules and centering the protein within the simulation box. The processed trajectories were used for subsequent analyses.

Protein backbone root mean square deviation (RMSD) was calculated to assess the overall structural stability of each protein during the simulation. Ligand RMSD was computed after fitting the trajectories to the protein backbone to evaluate the positional stability of TCDD within the binding pocket. Residue-level flexibility of the proteins was analyzed by calculating the root mean square fluctuation (RMSF) for each residue. All analyses were performed using standard GROMACS analysis tools.

### 2.10 qPCR validation

Total RNA was extracted from samples using the E.Z.N.A.® Total RNA Kit I (Omega Bio-tek) according to the standard protocol provided by the manufacturer. RNA quality assessment, including purity and concentration measurements, was conducted using a microplate reader. High-quality RNA (1 μg) was reverse transcribed into cDNA using the HiScript III RT SuperMix kit (Vazyme). Real-time quantitative PCR was performed on a LightCycler® 96 platform (Roche) using SYBR Green Master Mix (Vazyme) as the detection reagent. The PCR amplification program consisted of initial denaturation at 95 °C for 30 s, followed by 40 cycles of amplification reactions, with each cycle consisting of denaturation at 95 °C for 5 s and annealing/extension at 60 °C for 30 s. The relative expression levels of target genes PTGS1, PTGS2 were calculated using the 2^(-ΔΔCt) method with GAPDH as the internal reference gene. All samples were analyzed in triplicate. Primer sequences are listed as follows: PTGS1 Forward: 5′-TCC​ATG​TTG​GTG​GAC​TAT​GG-3′, Reverse: 5′-GTG​GTG​GTC​CAT​GTT​CCT​G-3'; PTGS2 Forward: 5′-CGG​TGA​AAC​TCT​GGC​TAG​ACA​G-3′, Reverse: 5′-GCA​AAC​CGT​AGA​TGC​TCA​GGG​A-3'.

### 2.11 Immunohistochemical (IHC) analysis

Formalin-fixed, paraffin-embedded synovial tissue sections (4 μm) were deparaffinized and rehydrated through graded alcohols. Antigen retrieval was performed using EDTA buffer (pH 9.0) under microwave heating. Endogenous peroxidase activity was blocked with 3% methanol-H_2_O_2_ for 25 min, followed by 3% BSA blocking for 30 min at room temperature. Sections were incubated overnight at 4 °C with rabbit primary antibodies against PTGS1 (1:200, Cat# GB112399, Servicebio, Wuhan, China) and PTGS2 (1:100, Cat# GB155672, Servicebio, Wuhan, China), respectively. After washing with PBS, HRP-conjugated goat anti-rabbit secondary antibody (S-vision polymer detection system, ready-to-use, Cat# G1302, Servicebio) was applied for 50 min at room temperature. Signals were visualized with DAB substrate and counterstained with hematoxylin. Images were captured using a light microscope (Nikon E100, Nikon Instruments, Tokyo, Japan). Staining intensity was semi-quantitatively assessed using Image-Pro Plus software.

### 2.12 Statistical analysis

All statistical analyses were performed using R software (version 4.3.1). Normality of data distribution was assessed using the Shapiro-Wilk test. For normally distributed continuous variables, Student’s t-test was used; otherwise, the Wilcoxon rank-sum test was applied. Multiple testing correction was performed using the Benjamini-Hochberg method. A two-sided P-value <0.05 was considered statistically significant unless otherwise specified. Receiver operating characteristic (ROC) curve analysis was performed to evaluate the diagnostic performance of individual genes using the pROC R package (Robin et al., 2011).

## 3 Results

### 3.1 Identification of OA-related differentially expressed genes

**FIGURE 1:**
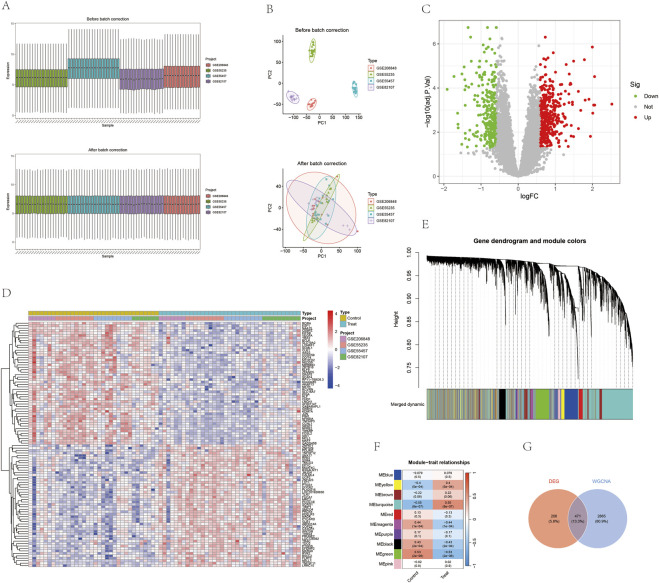
Identification of osteoarthritis-related genes through integrated bioinformatics analysis. **(A)** Box plots showing the expression distribution of four GEO datasets (GSE206848, GSE55235, GSE55457, and GSE82107) before (upper panel) and after (lower panel) batch correction. **(B)** PCA plots demonstrating sample clustering before (upper panel) and after (lower panel) batch correction. **(C)** Volcano plot displaying DEGs between OA and control samples. Red dots indicate significantly upregulated genes, green dots indicate significantly downregulated genes, and gray dots represent genes with no significant difference. **(D)** Heatmap of DEGs showing distinct expression patterns between control and OA (Treat) groups across different datasets. **(E)** Gene dendrogram and module assignment from WGCNA. **(F)** Module-trait relationship heatmap showing correlations between gene modules and clinical traits. Values represent Pearson correlation coefficients with p-values in parentheses. **(G)** Venn diagram illustrating the overlap between DEGs and WGCNA-identified OA-related genes, yielding 471 common genes for subsequent analysis.

To identify genes associated with osteoarthritis pathogenesis, we integrated four GEO datasets (GSE206848, GSE55235, GSE55457, and GSE82107) for comprehensive analysis. Prior to merging, substantial batch effects were observed among datasets, as evidenced by the heterogeneous expression distributions in box plots and distinct clustering patterns in PCA analysis (Figures 1A,B, upper panels). After applying batch correction using the ComBat algorithm, the expression levels were normalized across all datasets, and samples from different projects showed improved integration in PCA plots (Figures 1A,B, lower panels), indicating successful removal of technical variations while preserving biological signals. Quantitative evaluation using PVCA further confirmed this success. Before correction, the batch effect accounted for a dominant 82.17% of the total data variance, largely masking the true biological effect (17.82%). Following batch correction, the variance driven by the batch effect was drastically reduced to a negligible 0.06%, while the variance explained by the biological effect was highly enriched to 99.93%, demonstrating the robust elimination of non-biological technical noise.

Differential expression analysis identified a total of 677 DEGs between OA and control samples, comprising both upregulated and downregulated genes (Figure 1C). The heatmap visualization confirmed distinct expression patterns of these DEGs between the two groups (Figure 1D).

### 3.2 WGCNA identifies key gene modules associated with OA

To further explore gene co-expression patterns related to OA, we performed WGCNA on the integrated dataset. A total of 10 gene modules were identified based on expression similarity (Figure 1E). Module-trait relationship analysis revealed that several modules were significantly correlated with OA status (Figure 1F). Notably, the turquoise module showed the strongest positive correlation with OA (r = 0.55, p < 0.001), while the green module (r = 0.53, p < 0.001), magenta module (r = 0.44, p < 0.001), and black module (r = 0.43, p < 0.001) also demonstrated significant associations with the disease phenotype.

To obtain a more robust set of OA-related genes, we intersected the DEGs with genes from the significantly correlated WGCNA modules. As shown in the Venn diagram (Figure 1G), 471 overlapping genes (13.3%) were identified from 677 DEGs and 3,336 WGCNA module genes. These 3,542 consensus genes were OA-related genes and were used for subsequent network toxicology analysis with TCDD targets.

### 3.3 Identification of TCDD-OA common targets

**FIGURE 2:**
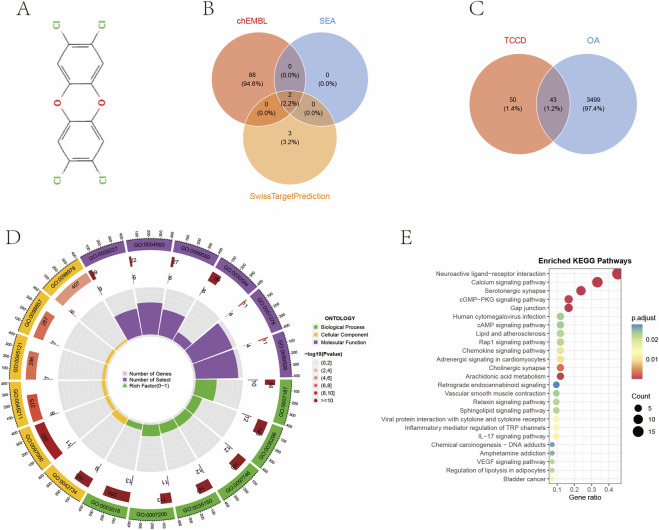
Network toxicology analysis of TCDD-related targets in osteoarthritis. **(A)** Chemical structure of TCDD. **(B)** Venn diagrams showing the identification of TCDD-OA intersection targets. **(C)** Venn diagram displaying the overlap between TCDD targets and OA-related genes from different sources. **(D)** Circular plot illustrating Gene Ontology (GO) enrichment analysis results of the 43 intersection targets, including Biological Process (green), Cellular Component (yellow), and Molecular Function (purple). The outer ring shows the number of genes, and the red circles indicate -log10(P-value). **(E)** Bubble plot depicting KEGG pathway enrichment analysis. The x-axis represents gene ratio, bubble size indicates gene count, and color gradient represents adjusted p-values.

TCDD is a persistent environmental pollutant with significant toxicological effects (Figure 2A). To investigate the potential toxic mechanisms of TCDD on OA, we first collected TCDD-related targets from multiple databases. A total of 88 targets were obtained from the ChEMBL database, while 3 targets were retrieved from the SwissTargetPrediction and 2 targets were retrieved from the SEA, with 2 targets overlapping between these three databases (Figure 2B). After removing duplicates, 93 TCDD-related targets were identified for subsequent analysis.

To identify the potential targets through which TCDD may exert toxic effects on OA, we intersected the 93 TCDD-related targets with OA-related genes. As shown in Figure 2C, 43 overlapping genes were identified, representing potential key targets mediating TCDD toxicity in the context of OA. These 43 intersection targets were subjected to functional enrichment analysis.

### 3.4 GO and KEGG enrichment analysis of intersection targets

GO enrichment analysis was performed to elucidate the biological functions of the 43 intersection targets (Figure 2D). In terms of Biological Process (BP), these targets were significantly enriched in G protein-coupled receptor signaling pathway, response to xenobiotic stimulus, cellular response to chemical stimulus, and positive regulation of cell communication. For Cellular Component (CC), the targets were predominantly located in plasma membrane, membrane raft, and receptor complex. Regarding Molecular Function (MF), the enriched terms included G protein-coupled receptor activity, transmembrane signaling receptor activity, and protein kinase activity.

KEGG pathway analysis revealed that the intersection targets were significantly enriched in multiple signaling pathways (Figure 2E). The most significantly enriched pathways included neuroactive ligand-receptor interaction, calcium signaling pathway, serotonergic synapse, cGMP-PKG signaling pathway, and gap junction. Additionally, several inflammation and immune-related pathways were identified, including IL-17 signaling pathway, chemokine signaling pathway, and cAMP signaling pathway. Notably, pathways involved in lipid metabolism (arachidonic acid metabolism, regulation of lipolysis in adipocytes) and vascular function (vascular smooth muscle contraction, VEGF signaling pathway) were also enriched, suggesting that TCDD may influence OA progression through multiple biological processes.

### 3.5 Machine learning-based identification of hub genes

**FIGURE 3:**
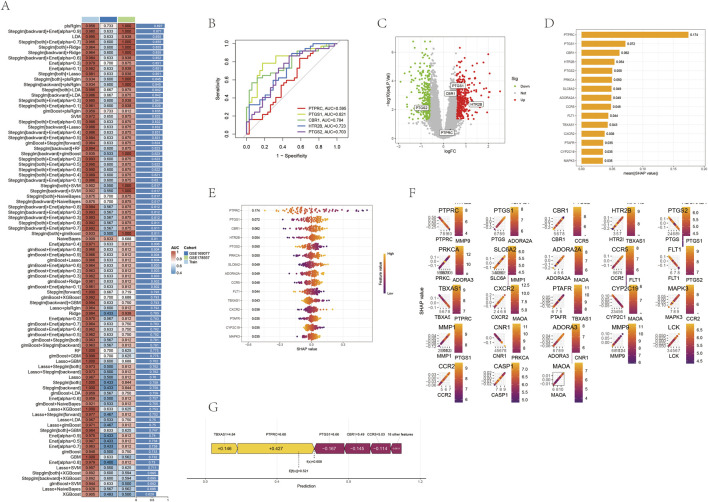
Machine learning-based identification and validation of hub genes. **(A)** AUC values were displayed across the training dataset and two validation cohorts (GSE169077 and GSE178557). The rightmost column shows the mean AUC. The top-performing algorithms are highlighted. **(B)** ROC curves validating the diagnostic performance of the identified hub genes (PTPRC, PTGS1, CBR1, HTR2B, and PTGS2) in distinguishing OA samples from controls. **(C)** Volcano plot highlighting the expression patterns of the five hub genes among all differentially expressed genes. Red dots indicate upregulated genes, green dots indicate downregulated genes, and hub genes are labeled. **(D)** Bar plot showing the mean absolute SHAP (SHapley Additive exPlanations) values for each feature, indicating the relative importance of genes in the prediction model. **(E)** Correlation matrix showing pairwise Spearman correlations among the hub genes. Each scatter plot displays the relationship between two genes, with correlation coefficients shown **(F)** SHAP summary plot displaying the distribution of SHAP values for each hub gene across samples from two validation cohorts (GSE169077 and GSE178557). Color indicates feature value (red: high, blue: low). **(G)** SHAP waterfall plot illustrating the contribution of individual features to a single prediction, demonstrating how each gene contributes to the final prediction outcome.

To identify the most critical genes from the 43 TCDD-OA intersection targets, we employed an integrative machine learning approach combining 127 algorithm combinations. The performance of each algorithm combination was evaluated using AUC across the training cohort and two independent validation cohorts (GSE169077 and GSE178557) (Figure 3A). Based on comprehensive evaluation, the optimal model was selected, which identified five hub genes: PTPRC (protein tyrosine phosphatase receptor type C), PTGS1 (prostaglandin-endoperoxide synthase 1), CBR1 (carbonyl reductase 1), HTR2B (5-hydroxytryptamine receptor 2B), and PTGS2 (prostaglandin-endoperoxide synthase 2). To explicitly evaluate the stability of our machine learning framework and mitigate potential overfitting risks, we further conducted a feature selection frequency analysis. While the initial framework comprised 127 theoretical algorithmic combinations, 89 combinations successfully converged and yielded stable predictive features across all cohorts. Within these 89 validated models, the identified hub genes demonstrated robust stability. Specifically, PTGS1 achieved a perfect selection frequency of 100%, followed by PTPRC (59.55%), PTGS2 (56.18%), CBR1 (53.93%), and HTR2B (20.22%). This robust frequency distribution quantitatively validates that our identified biomarkers are stable core features extracted from diverse computational logics, effectively minimizing the risk of single-model overfitting.

The diagnostic value of the five hub genes was validated using ROC curve analysis (Figure 3B). All hub genes demonstrated good discriminatory ability between OA and control samples. PTGS1 showed the highest diagnostic accuracy with an AUC of 0.821, followed by CBR1 (AUC = 0.784), HTR2B (AUC = 0.723), PTGS2 (AUC = 0.703), and PTPRC (AUC = 0.595). The volcano plot further confirmed that these hub genes exhibited significant differential expression in OA samples (Figure 3C). Specifically, PTGS1, CBR1, and HTR2B were upregulated, whereas PTGS2 was downregulated, and PTPRC showed no significant change.

### 3.6 SHAP analysis reveals feature importance

To interpret the machine learning model and understand the contribution of each gene to OA prediction, we performed SHAP analysis. The mean absolute SHAP values indicated that PTGS1 had the highest contribution to the model (mean |SHAP| = 0.072), followed by CBR1 (0.062), HTR2B (0.054), and PTGS2 (0.050) (Figure 3D).

To explore the potential functional relationships among the five hub genes, we performed pairwise correlation analysis (Figure 3E). The SHAP summary plot revealed distinct patterns for each hub gene across two independent validation cohorts (GSE169077 and GSE178557) (Figure 3F). The results revealed several significant correlations among hub genes. Notably, PTGS1 and PTGS2, both encoding prostaglandin-endoperoxide synthases, showed positive correlation, suggesting their coordinated involvement in arachidonic acid metabolism. Higher expression of PTGS1, CBR1, and HTR2B was associated with positive SHAP values (predicting OA), while lower expression of PTPRC contributed to OA prediction. The waterfall plot demonstrated how individual features collectively contributed to a specific prediction, with TBXAS1, PTPRC, PTGS1, CBR1, and CCR5 showing the most substantial contributions (Figure 3G).

### 3.7 Comprehensive immune infiltration analysis reveals distinct hub gene–immune microenvironment interactions

**FIGURE 4:**
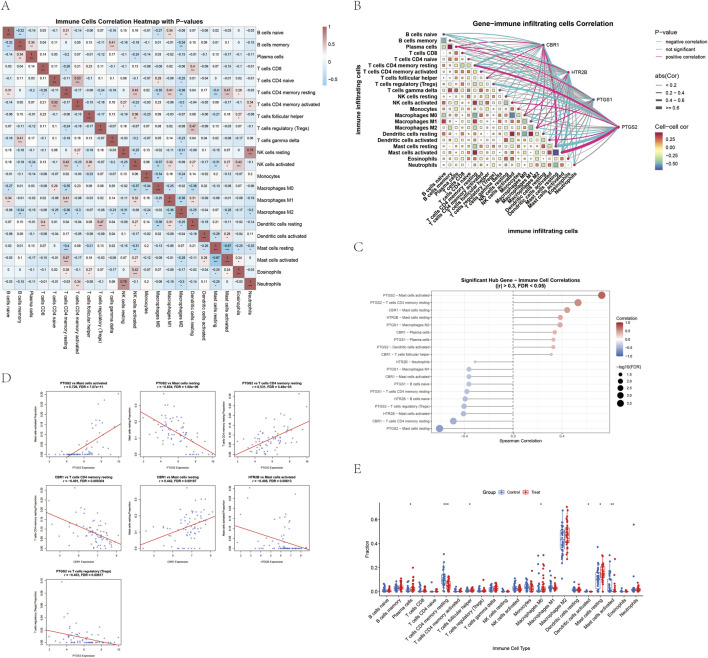
Comprehensive immune infiltration analysis of hub genes in OA. **(A)** Correlation network among immune cell types in OA. Heatmap depicts Spearman correlations among 22 immune cell types estimated by CIBERSORT in OA samples. Red indicates positive correlation; blue indicates negative correlation. Asterisks denote statistical significance (*p < 0.05, **p < 0.01, ***p < 0.001). **(B)** Hub gene–immune cell correlation analysis. Heatmap shows Spearman correlation coefficients between the expression levels of four hub genes (PTGS1, CBR1, HTR2B, PTGS2) and the relative proportions of 22 immune cell types. Red indicates positive correlation, blue indicates negative correlation. Color intensity corresponds to the absolute correlation coefficient. **(C)** Significant hub gene–immune cell correlations. Lollipop plot displays all significant gene–immune cell pairs (|r| > 0.3, FDR <0.05). The x-axis represents Spearman correlation coefficient; the y-axis shows gene–immune cell pairs sorted by correlation strength. Dot size indicates −log10(FDR). **(D)** Representative scatter plots of significant gene–immune cell pairs. Six significant correlations are displayed: PTGS2 vs. Mast cells activated (r = 0.726, FDR <0.001), PTGS2 vs. Mast cells resting (r = −0.604, FDR <0.001), PTGS2 vs. T cells CD4 memory resting (r = 0.531, FDR <0.001), CBR1 vs. T cells CD4 memory resting (r = −0.491, FDR <0.001), CBR1 vs. Mast cells resting (r = 0.442, FDR <0.001), and HTR2B vs. Mast cells activated (r = −0.408, FDR <0.001). **(E)** Differential immune cell infiltration between control and OA groups. Box plots compare the estimated proportions of 22 immune cell types between control (n = 37) and OA (n = 34) samples. Statistical significance was assessed using Wilcoxon rank-sum test (*p < 0.05, **p < 0.01, ***p < 0.001).

To systematically investigate the immune microenvironment associated with TCDD-induced osteoarthritis, we performed integrated immune infiltration analysis focusing on the four hub genes (CBR1, HTR2B, PTGS1, PTGS2). First, we examined the interrelationships among 22 immune cell types in OA samples, revealing a coordinated yet compartmentalized immune network characterized by strong positive correlations within myeloid lineage cells and negative correlations between T-cell subsets and certain innate immune cells (Figure 4A). Next, we assessed the associations between hub gene expression and immune cell infiltration patterns (Figure 4B). CBR1 was strongly associated with activated dendritic cells (r = 0.45, p < 0.001) and M1 macrophages (r = 0.39, p = 0.005), indicating a potential role in antigen presentation and pro-inflammatory responses. HTR2B demonstrated the most extensive immune correlations, with positive associations with regulatory T cells (Tregs; r = 0.48, p < 0.001) and M2 macrophages (r = 0.43, p = 0.001), suggesting its involvement in immune regulation. PTGS1 showed significant positive correlations with neutrophils (r = 0.38, p = 0.006) and M0 macrophages (r = 0.42, p = 0.002), suggesting its involvement in innate immune activation. PTGS2 exhibited negative correlations with CD8^+^ T cells (r = −0.41, p = 0.003) and natural killer cells (r = −0.36, p = 0.009), potentially reflecting an immunosuppressive role in OA.

To identify the most robust associations, we performed systematic correlation analysis between hub genes and immune cells, identifying 19 significant gene–immune cell pairs (|r| > 0.3, FDR <0.05) (Figure 4C). The strongest positive correlation was observed between PTGS2 and activated mast cells (r = 0.726, FDR <0.001), while the strongest negative correlation was between PTGS2 and resting mast cells (r = −0.604, FDR <0.001). Scatter plots of six representative pairs visually confirmed these linear relationships (Figure 4D). Finally, we compared immune cell proportions between OA and control samples, revealing significant alterations in the OA immune microenvironment, including increased proportions of M0 macrophages (p < 0.001), activated dendritic cells (p < 0.01), and regulatory T cells (p < 0.05), alongside decreased proportions of CD8^+^ T cells (p < 0.01) and resting mast cells (p < 0.05) (Figure 4E). These findings collectively suggest that TCDD-associated hub genes are embedded within an immune context characterized by enhanced innate immune activation, impaired cytotoxic responses, and increased regulatory mechanisms.

### 3.8 Molecular dynamics simulations reveal the stability of TCDD – protein interactions

**FIGURE 5:**
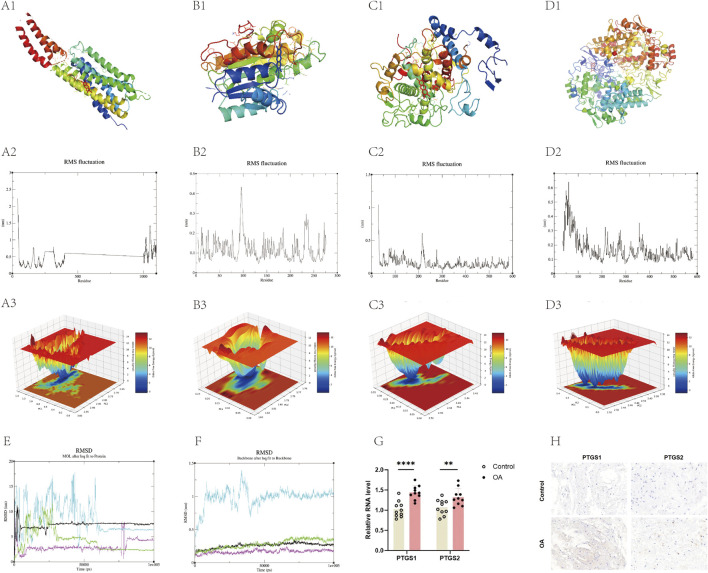
Molecular dynamics simulation analysis of TCDD binding to four hub proteins. **(A–D)** Representative three-dimensional structures of four hub proteins in complex with TCDD after MD simulations: **(A)** HTR2B, **(B)** CBR1, **(C)** PTGS1, and **(D)** PTGS2. Proteins are shown in cartoon representation colored by secondary structure, and TCDD is displayed as stick representation. **(A2–D2)** RMSF profiles of protein residues for each protein–TCDD complex during MD simulations. RMSF values reflect residue-level flexibility, with higher fluctuations mainly observed in terminal regions and solvent-exposed loops **(A3–D3)** FELs of the four protein–TCDD complexes projected onto the first two principal components (PC1 and PC2) obtained from principal component analysis (PCA) of the MD trajectories. Color gradients indicate free energy levels (kcal/mol), where blue regions represent low-energy, stable conformational states and red regions represent high-energy states. **(E)** RMSD of protein backbones for all four protein–TCDD complexes over the simulation time, illustrating the overall structural stability of each protein upon ligand binding. **(F)** RMSD of TCDD relative to the protein binding site for all four complexes, reflecting the positional stability of the ligand during MD simulations. **(G)** RT-qPCR validation of PTGS1 and PTGS2 mRNA expression levels in OA samples and healthy controls (n = 10 per group) **(H)** Representative IHC staining images of PTGS1 and PTGS2 protein expression in synovial tissue sections from OA patients and healthy controls. (*p < 0.05, **p < 0.01, ***p < 0.001).

Prior to evaluating dynamic stability, molecular docking was performed to assess the binding affinities between TCDD and the four identified hub proteins (Figures 5A1–D1). The results revealed favorable binding potentials for all targets, with binding affinities of −8.7 kcal/mol for PTGS2, −8.2 kcal/mol for CBR1, −7.8 kcal/mol for PTGS1, and −6.5 kcal/mol for HTR2B. To further investigate the dynamic stability of these protein–ligand complexes, MD simulations were performed. RMSF analyses confirmed that TCDD binding restricted local residue mobility within the binding pockets without compromising global protein flexibility (Figures 5A2–D2). Structural descriptors, including free energy landscapes (Figures 5A3–D3) and protein backbone RMSD (Figure 5E), indicated that all four complexes rapidly reached equilibrium and converged toward energetically favorable conformational states.

Following a comprehensive evaluation considering the machine learning selection frequency, the dynamic binding consistency in MD simulations (Figure 5F), and the established clinical significance in OA pathology, we prioritized PTGS1 and PTGS2 for subsequent experimental validation. RT-qPCR analysis confirmed that the relative mRNA expression levels of these targets were in full accordance with our bioinformatics predictions (Figure 5G). IHC staining demonstrated the upregulation and specific localization of the prioritized targets in the OA tissues compared to the healthy controls (Figure 5H).

## 4 Discussion

OA is a multifactorial degenerative joint disease characterized by progressive cartilage destruction, subchondral bone remodeling, and synovial inflammation (Martel-Pelletier et al., 2016). While mechanical stress and aging have been traditionally considered primary etiological factors, accumulating evidence suggests that environmental pollutants, particularly persistent organic pollutants, may contribute to OA pathogenesis (Berenbaum et al., 2018). TCDD, the most toxic congener of polychlorinated dibenzo-p-dioxins, has been epidemiologically associated with arthritis in exposed populations, including the Yucheng and Yusho cohorts (Guo et al., 1999). However, the molecular mechanisms underlying TCDD-induced joint damage remain incompletely understood. In this study, we employed an integrated network toxicology approach combining bioinformatics analysis and machine learning to systematically investigate the potential molecular targets and pathways through which TCDD may contribute to OA development.

Our analysis identified 3542 OA-related genes through the integration of differential expression analysis and WGCNA from four GEO datasets. Subsequently, 43 common targets were identified at the intersection of OA-related genes and TCDD targets predicted from ChEMBL, SwissTargetPrediction and SEA databases. Functional enrichment analysis revealed that these targets were significantly enriched in pathways related to inflammation, G protein-coupled receptor signaling, arachidonic acid metabolism, and calcium signaling. Through a comprehensive machine learning framework comprising 127 algorithm combinations, four hub genes (PTGS1, CBR1, HTR2B, and PTGS2) were identified with robust diagnostic performance across multiple validation cohorts. Molecular docking analysis confirmed favorable binding affinities between TCDD and all four hub proteins, with binding energies ranging from −7.7 to −8.7 kcal/mol.

PTGS2, also known as cyclooxygenase-2 (COX-2), demonstrated the strongest binding affinity with TCDD (−8.7 kcal/mol) in our molecular docking analysis. PTGS2 is an inducible enzyme that catalyzes the rate-limiting step in prostaglandin biosynthesis, converting arachidonic acid to prostaglandin H2, which is subsequently converted to prostaglandin E2 (PGE2) (Smith et al., 2000). In OA, elevated COX-2 expression has been consistently observed in chondrocytes, synoviocytes, and subchondral bone osteocytes (Hardy et al., 2002). PGE2 is a key mediator of OA pathology, promoting cartilage degradation through matrix metalloproteinase activation, inhibiting proteoglycan synthesis, and enhancing inflammatory cytokine production (Martel-Pelletier et al., 2003). Genome-wide association studies have identified PTGS2 gene polymorphisms as significant risk factors for hip and knee OA (Schneider et al., 2011). Furthermore, elevated COX-2 expression in subchondral bone osteocytes has been shown to induce spontaneous OA in mouse models, and COX-2 inhibition effectively attenuated cartilage degeneration (Tu et al., 2019). The strong binding affinity between TCDD and PTGS2 suggests that TCDD may exacerbate OA progression through direct modulation of prostaglandin synthesis pathways. Notably, although PTGS2 appeared downregulated in our transcriptomic analysis, both qPCR and IHC validation confirmed its elevated expression in OA synovial tissue, consistent with the well-established role of COX-2 in OA pathogenesis. This discrepancy likely reflects a limitation of the GEO reference samples, in which “healthy controls” were frequently obtained from patients undergoing surgery for acute joint injuries, whose synovial tissue may already exhibit COX-2 activation due to acute inflammatory responses, thereby confounding the comparative analysis.

PTGS1 (prostaglandin-endoperoxide synthase 1, also known as COX-1) showed the highest diagnostic accuracy among the hub genes (AUC = 0.821). Unlike PTGS2, PTGS1 is constitutively expressed and primarily responsible for maintaining physiological prostaglandin production (Smith and Langenbach, 2001). In cartilage, COX-1 expression is typically suppressed during OA progression, while COX-2 expression is enhanced, suggesting a shift from homeostatic to inflammatory prostaglandin synthesis (Fukai et al., 2012). Interestingly, studies have demonstrated that both COX-1 and COX-2 contribute to PGE2 production in cartilage, with differential regulation during disease progression (Amin et al., 1997). The identification of both PTGS1 and PTGS2 as hub genes indicates that TCDD may disrupt the delicate balance between constitutive and inducible prostaglandin synthesis, potentially accelerating the transition from healthy to osteoarthritic cartilage.

CBR1 (carbonyl reductase 1) demonstrated good diagnostic performance (AUC = 0.784) and showed significant binding affinity with TCDD (−8.2 kcal/mol). CBR1 is an NADPH-dependent oxidoreductase belonging to the short-chain dehydrogenase/reductase superfamily, with broad specificity for carbonyl compounds including quinones, prostaglandins, and xenobiotics (Oppermann, 2007). A critical function of CBR1 is protecting cells against oxidative stress by inactivating highly reactive lipid aldehydes such as 4-hydroxynonenal (4-HNE) and 4-oxonon-2-enal, which accumulate during oxidative damage and can modify proteins and DNA (Mao et al., 2021). Oxidative stress is a well-established contributor to OA pathogenesis, driving chondrocyte senescence, matrix degradation, and inflammatory responses (Loeser et al., 2016). Additionally, CBR1 has been shown to modulate inflammatory responses by inhibiting NF-κB and MAPK signaling pathways, suppressing COX-2 expression and PGE2 production (Kim et al., 2015). The interaction between TCDD and CBR1 may compromise the cellular defense against oxidative stress, thereby exacerbating oxidative damage in articular cartilage.

HTR2B (5-hydroxytryptamine receptor 2B), encoding the serotonin receptor 2B, emerged as a novel hub gene linking TCDD exposure to OA pathogenesis. HTR2B is a G protein-coupled receptor that mediates diverse physiological functions including cardiovascular regulation, bone metabolism, and immune modulation (Berger et al., 2009). Importantly, HTR2B has been shown to be required for normal osteoblast function and proliferation, and for maintaining normal bone density (Collet et al., 2008). The 5-HT2B receptor also plays critical roles in immune cell polarization, particularly in macrophages, where its activation promotes the anti-inflammatory M2 phenotype while suppressing the pro-inflammatory M1 phenotype (de las Casas-Engel et al., 2013). In the context of joint disease, serotonin receptor expression on fibroblasts has been associated with inflammation in both rheumatoid arthritis and osteoarthritis synovium (Xiang et al., 2024). The binding of TCDD to HTR2B may disrupt serotonin-mediated regulation of bone metabolism and immune cell function, contributing to the subchondral bone changes and synovial inflammation characteristic of OA.

The pathogenic effects of TCDD on cartilage have been demonstrated in multiple experimental models. TCDD has been shown to induce chondrocyte apoptosis through reactive oxygen species (ROS)-dependent activation of PKC-δ and caspase-3 pathways (Lee and Yang, 2010). In zebrafish embryos, TCDD exposure reduces chondrocyte size and number in craniofacial cartilages, with downregulation of sox9b, a master transcription factor for chondrogenesis (Burns et al., 2015). These effects are mediated primarily through the aryl hydrocarbon receptor (AhR), which is widely expressed in growth plate cartilage and synovial tissue (Alhamad et al., 2022). Upon binding to AhR, TCDD activates transcription of target genes including CYP1A1 and pro-inflammatory cytokines such as IL-1β, IL-6, and IL-8 through NF-κB and ERK signaling cascades (Vogel and Matsumura, 2009). Epidemiological studies have established positive associations between dioxin exposure and arthritis prevalence in the general population, with the Yucheng and Yusho poisoning cohorts showing significantly elevated rates of arthritis decades after exposure (Meng et al., 2024). Our findings extend these observations by identifying specific molecular targets through which TCDD may exert its arthritogenic effects.

Functional enrichment analysis revealed that the TCDD-OA common targets were significantly enriched in several pathways relevant to joint pathology. The arachidonic acid metabolism pathway, involving both PTGS1 and PTGS2, is central to prostaglandin-mediated inflammation and cartilage degradation in OA (Korotkova and Jakobsson, 2011). The neuroactive ligand-receptor interaction pathway, encompassing HTR2B and other G protein-coupled receptors, suggests involvement of neurotransmitter signaling in TCDD-induced joint pathology. The IL-17 signaling pathway has emerged as a critical mediator of both osteoarthritis and rheumatoid arthritis, promoting cartilage destruction and bone erosion (Mimpen and Snelling, 2019). The calcium signaling pathway is essential for chondrocyte function and mechanotransduction, and its dysregulation contributes to OA progression (Guilak, 2011). The convergence of these pathways on the identified hub genes provides a systems-level understanding of how TCDD exposure may contribute to OA development through multiple interconnected mechanisms.

Several limitations of this study should be acknowledged. First, this study was based on bioinformatics analysis of publicly available transcriptomic data, and the predicted interactions between TCDD and hub proteins require experimental validation through *in vitro* binding assays and functional studies. Second, while our molecular docking and molecular dynamics simulations demonstrate high binding affinities between TCDD and the identified hub proteins, a cautious interpretation of the affinity-activity relationship is strictly warranted. High binding affinity (computational prediction) does not intrinsically equate to functional biological activity (experimental confirmation). Current structural simulations cannot definitively determine whether the binding of TCDD exerts agonistic, antagonistic, or functionally silent effects on these target proteins. Furthermore, these *in silico* models do not fully account for the complex cellular context, including post-translational modifications or competitive binding in the physiological microenvironment. Third, although our machine learning framework integrated 127 algorithms and utilized feature selection frequency analysis to minimize overfitting, high-dimensional transcriptomic screening intrinsically carries algorithmic biases. Fourth, the cross-sectional nature of the analyzed GEO datasets inherently limits our ability to make definitive causal inferences regarding disease progression. Finally, the inherent uncertainties associated with database-driven target predictions emphasize that these computational models fundamentally serve as robust statistical hypotheses rather than confirmed biological realities. To address these computational limitations, future studies must prioritize actionable experimental validations. Specifically, *in vitro* validation should be conducted using primary chondrocytes treated with relevant TCDD concentrations (e.g., 0.1–10 nM) to monitor hub gene expression and cartilage degradation. Additionally, targeted functional studies, such as utilizing HTR2B knockout models or specific pharmacological inhibitors, are strictly warranted to establish causal relationships and elucidate the precise signaling mechanisms in OA pathogenesis.

In conclusion, this study employed an integrated network toxicology and machine learning approach to systematically investigate the potential molecular mechanisms underlying TCDD-induced exacerbation of osteoarthritis. Four hub genes (PTGS1, CBR1, HTR2B, and PTGS2) were identified as key molecular targets mediating the effects of TCDD on OA pathogenesis. These genes are involved in prostaglandin synthesis, oxidative stress response, immune cell function, and neurotransmitter signaling, suggesting that TCDD may contribute to OA development through multiple converging pathways. Molecular docking analysis confirmed favorable binding affinities between TCDD and all four hub proteins. These findings provide new insights into the environmental risk factors of OA and identify potential therapeutic targets for intervention strategies. Further experimental validation is warranted to confirm these computational predictions and to elucidate the detailed mechanisms of TCDD-induced joint pathology.

## Data Availability

The datasets presented in this study can be found in online repositories. The names of the repository/repositories and accession number(s) can be found in the article/supplementary material.
